# Innate adaptive immune cell dynamics in tonsillar tissues during chronic SIV infection

**DOI:** 10.3389/fimmu.2023.1201677

**Published:** 2023-08-21

**Authors:** Rajni Kant Shukla, Manuja Gunasena, Nicole Reinhold-Larsson, Michael Duncan, Amila Hatharasinghe, Samuel Cray, Krishanthi Weragalaarachchi, Dhanuja Kasturiratna, Thorsten Demberg, Namal P. M. Liyanage

**Affiliations:** ^1^ Department of Microbial Infection and Immunity, College of Medicine, Ohio State University, Columbus, OH, United States; ^2^ Department of Mathematics and Statistics, Northern Kentucky University, KY, Highland Heights, KY, United States; ^3^ Department of Pediatrics, Baylor College of Medicine, Houston, TX, United States; ^4^ Department of Veterinary Biosciences, College of Veterinary Medicine, Ohio State University, Columbus, OH, United States; ^5^ Infectious Diseases Institute, The Ohio State University, Columbus, OH, United States

**Keywords:** SIV, tonsils, NK, T cells, monocytes, NKB cells, CD161+T cells

## Abstract

HIV-infected patients are at higher risk of developing oral mucosal infection and Epstein–Barr virus (EBV)-associated B cell malignancies. However, the potential role of oral immunity in the pathogenesis of oral lesions is unknown. Tonsils are oral-pharyngeal mucosal-associated lymphoid tissues that play an important role in oral mucosal immunity. In this study, we investigated the changes of innate and adaptive immune cells in macaque tonsils during chronic SIV infection. We found significantly higher frequencies of classical monocytes, CD3+CD56+ (NKT-like) cells, CD3^+^CD4^+^CD8^+^ (DP), and CD161^+^ CD4 T cells in tonsils from chronic infected compared to naïve animals. On the contrary, intermediate monocytes and CD3^+^CD4^-^CD8^-^ (DN) cells were lower in chronic SIV-infected macaques. We further confirmed a recently described small B-cell subset, NKB cells, were higher during chronic infection. Furthermore, both adaptive and innate cells showed significantly higher TNF-α and cytotoxic marker CD107a, while IL-22 production was significantly reduced in innate and adaptive immune cells in chronic SIV-infected animals. A dramatic reduction of IFN-γ production by innate immune cells might indicate enhanced susceptibility to EBV infection and potential transformation of B cells in the tonsils. In summary, our observation shows that the SIV-associated immune responses are distinct in the tonsils compared to other mucosal tissues. Our data extends our understanding of the oral innate immune system during SIV infection and could aid future studies in evaluating the role of tonsillar immune cells during HIV-associated oral mucosal infections.

## Introduction

Although anti-retroviral treatment (ART) has reduced the morbidity and substantially decreased the mortality associated with HIV infections, severe forms of oral diseases such as periodontitis and dental caries have not been dropped in people living with HIV(PLWH) on ART (1, 2). Several studies suggest that PLWH develop immune reconstitution inflammatory syndrome (IRIS) that manifests as recurrent oral ulcers and parotid enlargement after ART initiation (3). In addition, some studies show oral malignancies, such as Epstein Barr Virus mediated transforming of B cells in tonsils, are higher among PLWH (4, 5). A recent study showed a connection between CD4 T cells and oral immune dysfunction in HIV infection (5, 6). However, the exact mechanism and etiology of these pathological conditions are poorly studied. Tonsils are secondary lymphoid organs belonging to the mucosal-associated lymphoid tissues, and tonsillar crypt epithelium is specialized for immune surveillance (7). In addition, the tonsils serve as a reservoir and site of replication for HIV (8). However, tonsillar innate and adaptive immune cell populations and their changes during chronic HIV infection are not well studied. The rhesus macaque SIV model is an important system for studying HIV pathogenesis. Li et al., studied the role of NK and ILCs in SIV-infected rhesus macaque oral mucosa, including tonsils in SIV-infected animals (9). We used tonsils from chronic SIV-infected rhesus macaques and healthy animals to investigate differences in innate and adaptive immune cell composition and function. Using an unbiased clustering approach based multivariant t-mixture model, we compared immune cell clusters between naïve and chronic SIV-infected macaques. We found eight statistically distinct immune clusters, mainly T, B, and NK cell subsets. Detailed analysis showed significantly elevated classical monocytes and higher TNF-α production in the intermediate monocyte population in chronic SIV-infected animals. Similar to previous reports (9), we observed higher cytotoxicity (CD107a) in NKp44^+^ cells. In addition, we found more NKT cells and reduced production of TNF-α in NKB-like cells in chronic SIV-infected animals. Although we did not see a significant alteration of CD4^+^ and CD8 T^+^ cells, we found a substantial loss of CD3^+^CD4^-^CD8^-^ (DN) cells in the tonsils of infected macaques. Furthermore, we found significantly more CD161^+^ CD4 T cells in chronic SIV infected animals, but their functional capacity to produce IFN-γ was significantly reduced.

## Materials and methods

### Ethics statement

All animal experiments were approved by Institutional Animal Care and Use Committees (IACUCs) prior to study initiation, including the NCI/NIH Animal Care and Use Committee and the Advanced BioScience Laboratories, Inc. (ABL) Animal Care and Use Committee. Rhesus macaques were maintained at the NCI Animal Facility (Bethesda, MD) under protocol VB012 and VB001. Following the challenge phase of the study, macaques were housed at ABL (Rockville, MD) under protocol AUP526. Each of these facilities is accredited by the Association for Assessment and Accreditation of Laboratory Animal Care (AAALAC) International. The standard practices closely follow recommendations made in the National Institutes of Health Guide for the Care and Use of Laboratory Animals. Animals were housed in accordance with the recommendations of the AAALAC Standards and with the recommendations in the Guide for the Care and Use of Laboratory Animals. Details of animal welfare and steps taken to ameliorate suffering were in accordance with the Guide for the Care and Use of Laboratory Animals and the recommendations of the Weatherall report on the use of NHPs in research, as approved by the relevant IACUCs.

### Animals and SIV infections

A total of 20 young Male (6 to 8 years old) Indian Rhesus macaque (Macaca mulatta), 10 SIV naïve, and 10 chronic SIVmac_251_ infected were included in this study. SIV naïve animal’s tonsils were obtained from the control arms of a previous, unpublished vaccine immunogenicity study. Control animals received saline intra-rectally during the challenge phase. None of these animals were exposed to SIV antigens. SIV-infected animals were obtained from a previously published study (10). chronic SIV infected macaques were challenged with repeated low doses of SIV_mac251_ intra-rectally, and tonsils were obtained when animals were sacrificed 48 weeks after primary SIV^+^ results.

### Cell collection and processing

Tonsils were all collected at necropsies and lymphocytes isolate using assays optimized in our laboratory and according to the published protocol (11). Briefly, using forceps, place tonsils in a large plastic petri dish and cover with 4°C HBSS. Tonsil cut into 3- to 10-mm fragments with scissors and push lymphoid cells through the mesh using the flat end of a 60-ml plastic syringe plunger. Rinse the remaining tissue two to three times with HBSS until clear. Transfer equal volumes of cell suspension from the petri dish to 50-ml centrifuge tubes. Add colder HBSS and process the remaining tissue fragments by repeating steps twice to free as many lymphocytes as possible from the stroma. After transferring all cells to centrifuge tubes, break up any cell clumps and suspend the cells by repeated pipetting. Underlay cell suspension with 10 ml Ficoll-Hypaque. Centrifuge 20 min in a Sorvall H-1000B rotor at 1800 rpm (1000 × *g*), room temperature. Accelerate slowly and decelerate without braking. Collect mononuclear cells from the interface and discard cell pellets containing fibroblasts and other cell debris.

### Flow cytometry cell staining and analysis

Tonsil single-cell suspensions were thawed, washed, and resuspended in R10 medium and incubated overnight at 37°C and 5% CO2 prior to staining for flow cytometry or activation for the intracellular staining. 2 million cells were stained with non-human primate cross-reactive fluorescent tag antibodies (**Supplemental Table 1**). LIVE/DEAD Aqua dye (Invitrogen) and isotype-matched controls and/or fluorescence-minus-one (FMO) controls were included in all assays. Acquisitions were made on a Cytek Aurora spectral flow cytometer (Cyteck Biosciences, CA) and analyzed using FlowJo version 10.6.1 (Becton Dickinson, Ashland, OR) for high dimensional data analysis following standardized workflow. The first step was integrating the flow cytometry marker data from all the infected and uninfected samples for implemented compensation and scaling using OMIQ (Dotmatics, Boston, MA). Then, Non-linear dimensionality reduction was performed using t-Distributed Stochastic Neighbor Embedding (tSNE) (12)to visualize multi-dimensional expression landscapes in two dimensions (2D). Next, cell subsets were visualized by overlaying defined immune subset information onto the 2D tSNE plots. Next, unbiased clustering was performed on the expression values of the lineage markers using the FlowSOM algorithm in OMIQ, which uses a self-organizing map followed by hierarchical consensus meta-clustering to detect cell populations. Finally, a reduced two-dimensional space was implemented to visualize unbiased clusters using the Uniform Manifold Approximation and Projection (UMAP) algorithm (13) in the OMIQ. The next step was to annotate cell subsets by visually investigating heat maps of median marker expressions across clusters and expressions of these markers in the UMAP space.

### Intracellular cytokine staining procedure (ICS)

To assess innate and adaptive immune cell function, Tonsillar (1 x10^6^ cells) were resuspended in RPMI 1640(Gibco) containing 10% FBS and either stimulated with PMA (50 ng/ml) and ionomycin (1 μg/ml) or cultured in medium alone. Anti-CD107a was added to each tube at a concentration of 20 μl/ml and, Golgiplug (brefeldin A) [Cat No: 555029, BD biosciences] and Golgistop (monensin) [Cat No: 554724, BD biosciences] were added at final concentrations of 6 μg/ml; then all samples were cultured for 12 h at 37°C in 5% CO2. After culture, samples were surface stained using markers to delineate live cells (LIVE/DEAD Aqua dye), leukocytes (CD45), and innate and adaptive immune cell populations (complete list of antibodies in the **Supplemental Table 1**). Cells were then permeabilized using Caltag Fix & Perm, and intracellular cytokine staining was performed for combinations of antibodies to measure IFN-γ, TNF-α, IL-22.

### Statistical analyses

All statistical and graphical analyses were performed using GraphPad Prism software (GraphPad Software, Inc., La Jolla, CA). The Mann-Whitney-Wilcoxon test was used to compare continuous factors between two groups. Correlation analyses were performed using the Spearman rank correlation method with exact permutation P value.

## Result

### Unique innate and adaptive immune cell composition in tonsils from naïve rhesus macaques

**Figure 1:**
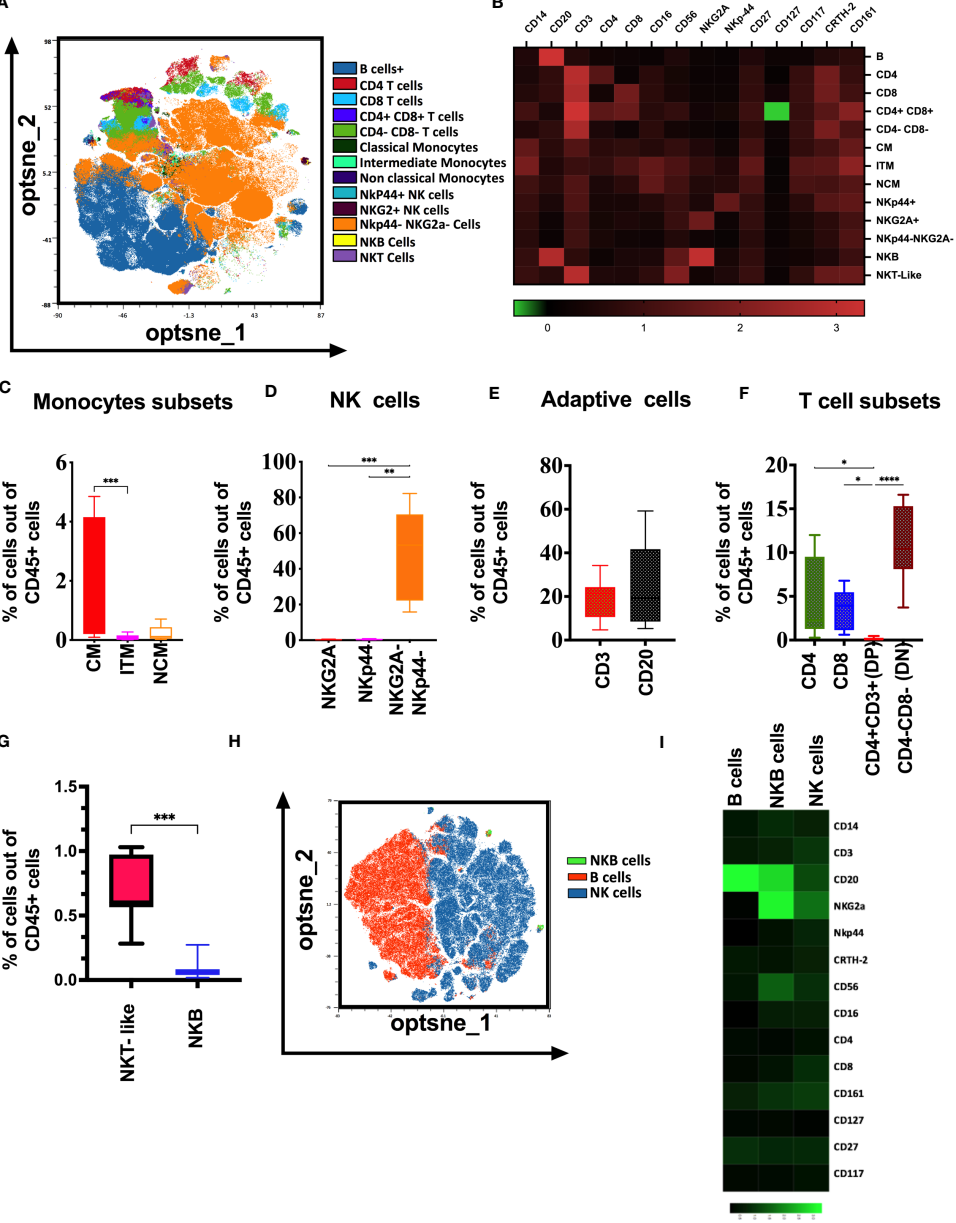
Immunologic map of naïve rhesus macaques’ tonsils. **(A)** T-distributed stochastic neighbor embedding (t-SNE) analysis of concatenated 10 naïve rhesus macaque’s tonsillar immune cell subsets gated on total viable CD45^+^ cells. **(B)** The heatmaps shows median marker intensities within each immune cell population. Percentage out of total lymphocytes of **(C)** monocyte subsets, **(D)** NK cell subsets, **(E)** adaptive cell subsets, **(F)** T cell subsets present in the healthy naïve rhesus macaque’s tonsils. **(G)** Distribution of NKT-like, and NKB cells in native macaques’ tonsils **(H)** t-SNE analysis showing selected B cell and NK cell surface receptor expression in B cells, NK cells and NKB cell cells in naïve macaques’ tonsils. **(I)** Relative expression of NKG2A, NKp44, CD16 and CD56 markers visualized over the t-SNE plots is displayed as a colorimetric scale of black (low expression) to green (high expression). One-way ANOVA were used for multiple parameter for comparisons; *p<0.05 **p< 0.01, ***p< 0.001, ****p-< 0.0001.

To elucidate the immune cell composition in adult rhesus macaques’ tonsils, we performed high dimensional flow cytometry on cryopreserved lymphocytes from tonsils. We first assessed the percentage of different innate and adaptive immune cells in tonsils using manual gating (**Supplemental Figure 1**) and unsupervised raw data clustering of single live CD45^+^ cells. We defined innate (monocytes and NK), adaptive (T and B cells), and unconventional (NKB) immune cells based on receptor expression and compared the abundance of each subset. We found that CD3^-^CD20^-^NKG2A^-^NKp44^-^ cells are predominant in macaque tonsils, followed by B cells (CD20^+^) and CD3^+^CD4^-^CD8^-^ (Double Negative) T cells respectively (**Figure 1**). Similar to peripheral blood, we identified three major monocyte populations, classical monocytes (CD14^++^CD16^-^), non-classical (CD14^+^CD16^++^) and intermediate (CD14^++^CD16^+^) monocytes. The classic subset was the dominant monocyte population in the tonsils (**Figure 1C**). We found that CD3^-^CD20^-^CD14^-^NKG2A^-^NKp44^-^ cells are more common than classical cytotoxic NK cells (CD3^-^CD20^-^ CD14^-^NKG2A^+^) and regulatory NK (CD3^-^CD20^-^ CD14^-^NKp44^+^) cells in tonsils from naïve animals (**Figure 1D**). B cells were more common than CD3 cells in the tonsils. However, the difference was not statistically significant (**Figure 1E**). CD4 and CD8 T cells were more common than CD3^+^CD4^+^ CD8^+^ T (DP) cells. However, DN cells (CD3^+^CD4^-^CD8^-^) were the dominant population of CD3^+^ cells in the tonsils (**Figure 1F**). Additionally, we found that NK-T-like cells, as well as the recently described B-cell population, termed NKB cells, present in the naïve macaque’s tonsils (**Figures 1G–I**). NKB cells express CD20 (B cell receptor) and NKG2A, NKp44, CD16, and CD56 receptors on their surface.

### Effect of SIV chronic infection on tonsillar immune cell dynamics

**Figure 2:**
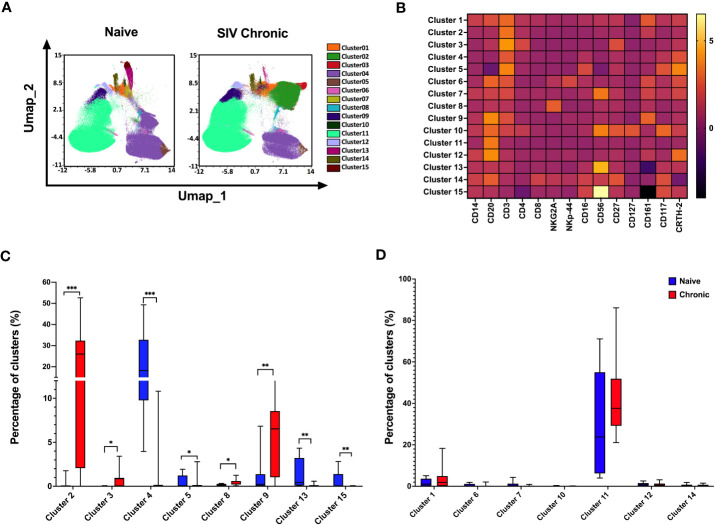
Differences in the composition of immune cell populations in tonsils from naïve and SIV chronic rhesus macaques. **(A)** A total of 1000000 mononuclear cells from naïve rhesus macaques (n=10) and SIV chronic (n=10) were clustered using an unbiased multivariate t mix model, which defined 15 sub-clusters that reflect statistically distinct cell populations and visualization of the relative similarities of each cell and cell cluster on the two-dimensional UMAP space with 10% down sampling. **(B)** Clusters by marker heatmap characterizing the expression patterns of individual clusters**. (C, D)** Cluster frequencies of naïve vs healthy animals’ tonsils samples. The P value was calculated using Wilcoxon rank-sum test *p<0.05, **p < 0.01, ***p < 0.001.

Next, we utilized an unbiased clustering approach based on a multivariant t-mixture model to analyze our high-dimensional spectral flow cytometric data comparing cell populations from naïve to chronically SIV infected animal’s tonsils. We identified eight distinct immune cell clusters out of 15 that we visualized in two dimensions using the UMAP algorithm (**Figure 2**). Using clustered heatmap analysis. We correlated expression intensity with clusters to annotate T cells (clusters 1,2,3,4,5), B cells (clusters 11, 12), unconventional cells (clusters 6: T cells expressing B cells receptor CD20 and NK receptor NKp44, clusters 7: NKT-like cells (CD3+CD56+CD161+CRTH2+), NK cells (clusters 8, 13,15), other immune cells (cluster 9: CD20+CD3+CD161+), cluster10: CD20+CD3+CD4+CD56+CD161+, cluster14: CD20+CD8+CD27+). Comparing immune population dynamics, we found significant higher CD161^+^ CD4 T cells (cluster 2), CD27^+^ CD4 T cells (cluster 3), NKG2A^+^ NK cells (cluster 8), and CD161^+^ CD3^+^CD20^+^ cells (cluster 9) in chronic SIV infected macaques. Conversely, we observed significantly lower CRTH2^+^ NKp44^+^ cells (cluster 4), CD56^+^NKG2A^+^ NK cells (cluster 13), and CD117^+^ CD27^+^ innate cells (cluster 15) in chronic SIV compared to uninfected animals.

### Dynamics of tonsillar monocyte subsets during chronic SIV infection in rhesus macaque

**Figure 3:**
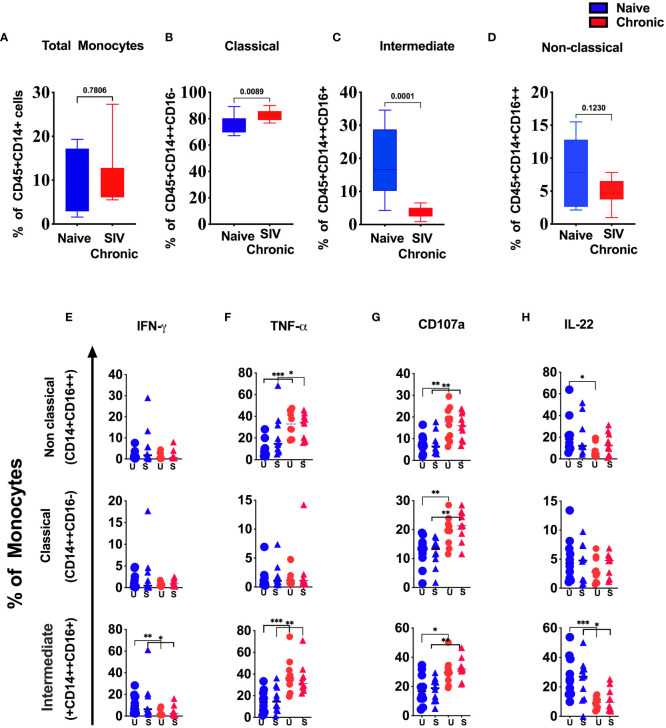
Changes in tonsillar monocytes subsets during chronic SIV infection in rhesus macaque. Frequency of **(A)** total, **(B)** classical, **(C)** intermediate and **(D)** non-classical monocytes in naïve and chronic SIV-infected rhesus macaques’ tonsils. Production of **(E)** IFN-γ, **(F)** TNF-α, **(G)** CD107a and **(H)** IL-22 in non-classical, classical and intermediate monocytes in naïve vs chronic macaque’s PMA/Ionomycin stimulated (S) and unstimulated (US) tonsillar monocytes. *p < 0.05, **p < 0.01, ***p < 0.001. Non-parametric T test used for p-value calculation. Each group have 10 animals.

It is well-established that HIV infection impacts monocytes subpopulations (14). Thus, we compare the frequency and functional difference of the monocyte populations between tonsils from naïve and chronic SIV infected animals (**Figure 3**). We observed a slight reduction of total monocytes (**Figure 3A**), with classical monocytes (CM) being the dominant population in infected tonsils (**Figure 3B**). In contrast, inflammatory monocytes (intermediate/ITM) were significantly depleted when compared to healthy animals (**Figure 3C**). Concurrently, we found lower IFN-γ production in intermediate monocytes (**Figure 3E**) but not in the other two monocytes subsets. In contrast to IFN-γ, the production of TNF-α (**Figure 3F**) was higher in non-classical and intermediate monocytes in tonsils from SIV-infected animals. We further found a significantly higher CD107a (**Figure 3G**) in all three subsets indicating a more activation, potentially skewed cytokine profile, in SIV infection. While we found considerable IL-22 production by monocyte subsets in tonsillar monocytes from uninfected animals, IL-22 expression in non-classical and intermediate monocytes were significantly reduced in chronic SIV infected animals (**Figure 3H**).

### Chronic SIV infection modulates tonsillar NK/ILC subsets in the rhesus macaque

**Figure 4:**
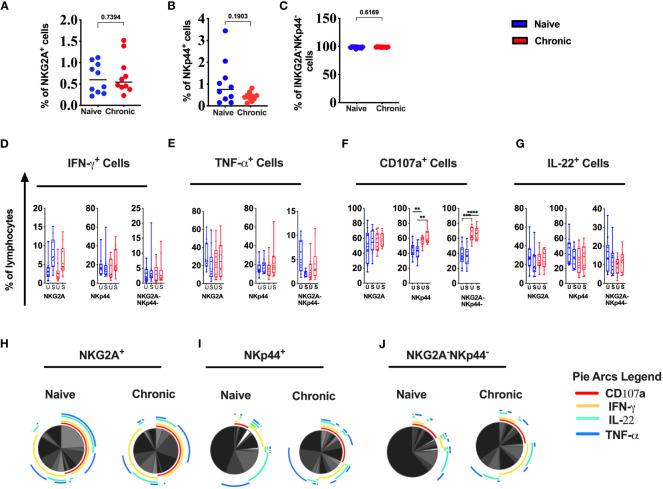
Chronic SIV infection modulates the function of tonsillar NK cells in rhesus macaques. Frequencies of **(A)** NKG2A^+^, **(B)** NKp44^+^ & **(C)** NKG2A^-^NKp44^-^ cells in naive and chronic SIV infected macaques. Tonsillar mononuclear cells were stimulated with PMA/ionomycin for 12h and production of IFN-γ **(D)**, TNF-α **(E)**, CD107a **(F)** expression and IL-22 **(G)** production were measured in NK cell subsets in naive and chronically SIV-infected macaques. **(H-J)** Multifunctional NK cell subsets were identified by SPICE software (v.5.22). Mann–Whitney U-tests were used for naïve vs.-SIV comparisons and Wilcoxon matched pairs tests were used to compare NK cells and ILCs; **p < 0.01, ****p < 0.0001.

We and other previously described three major types of NK cell subsets in rhesus macaque mucosal tissues (10, 15). All three NK cell subsets were present in tonsils from naïve animals (**Supplemental Figure 1**). There was a reduction of NKp44^+^ (NKp44^+^ ILC) cells in SIV-infected tonsils. Still, the difference was not statistically significant (**Figure 4B**). NKG2A+ NK (**Figure 4A**) and NKp44-NKG2A- (DN) cells (**Figure 4C**) did not differ between naïve and tonsils from SIV-infected animals. In addition, we measured IFN-γ, TNF-α, IL-22 and CD107a in NK cell subsets (**Figures 4D–G**). We found no statistically significant difference in IFN-γ, TNF-α, and IL-22 secretion between NK cell populations from naïve and chronic SIV-infected animals. However, CD107a (degranulation) was significantly higher at baseline (unstimulated) as well as post-stimulation in NKp44^+^ cells and NKp44^-^ NKG2A^-^ cells (**Figure 4F**) in the tonsils of chronically SIV-infected animals. SPICE analysis (**Figures 4H–J**) showed higher multifunctionality in NKp44^+^cells (**Figure 4I**) and NKG2A^-^NKp44^-^(DN) cells (**Figure 4J**) from SIV-infected tonsils.

### NKT-like cells and NKB cells in macaque tonsils during SIV infection

**Figure 5:**
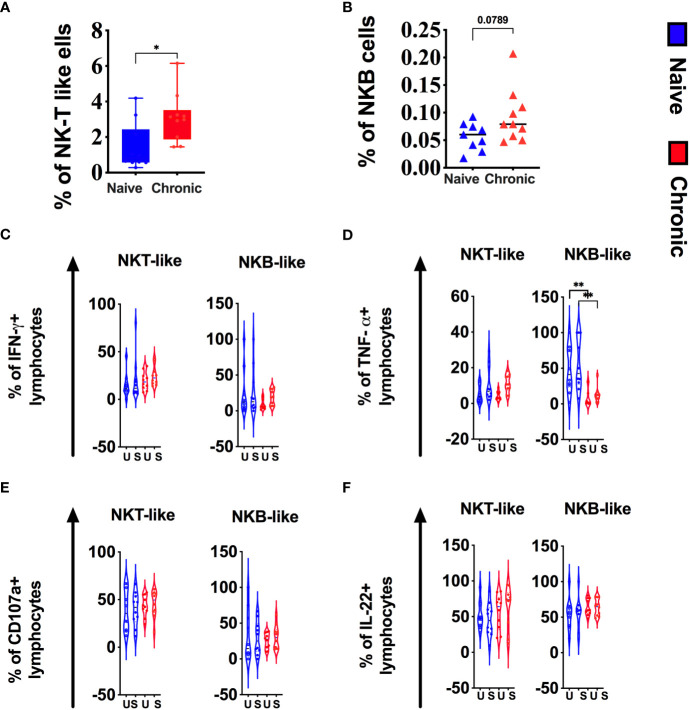
Chronic SIV infection alters NKT-like and NKB cells in tonsils of rhesus macaques. Frequencies of NKT-like **(A)** and NKB **(B)** cells in naive and chronic SIV infected macaques. Tonsillar mononuclear cells were stimulated with PMA/ionomycin for 12h and production of **(C)** IFN-g, **(D)**TNF-a, (E)CD107a expression and **(F)** IL-22 production were measured in NKT-like and NKB subsets in naive and chronically SIV-infected macaques. Mann–Whitney U-tests were used for nanaïve vs. SIV+ comparisons and Wilcoxon matched pairs tests were used to compare ILC subsets; *P < 0.05; **P < 0.01.

NKT–like cells (CD3^+^CD56^+^) and NKB cells (CD20^+^NKG2A^+^) are poorly described but might play a role in bridging the innate and adaptive immune response. Alternatively, both cell types might higher as part of immune dysregulation/exhaustion in chronic disease. In this study, we found a significantly higher level of NKT-like cells in tonsils from chronically SIV macaques (**Figure 5A**). Although not statistically significant, NKB cells (**Figure 5B**) were also higher in infected animals. We found no difference in cytokine production by NKT cells between naïve and infected animals (**Figure 5C, E, F**). However, NKB cells from infected animals produced significantly lower levels of TNF-α (**Figure 5D**).

### Tonsillar T cell subsets during chronic SIV infection

**Figure 6:**
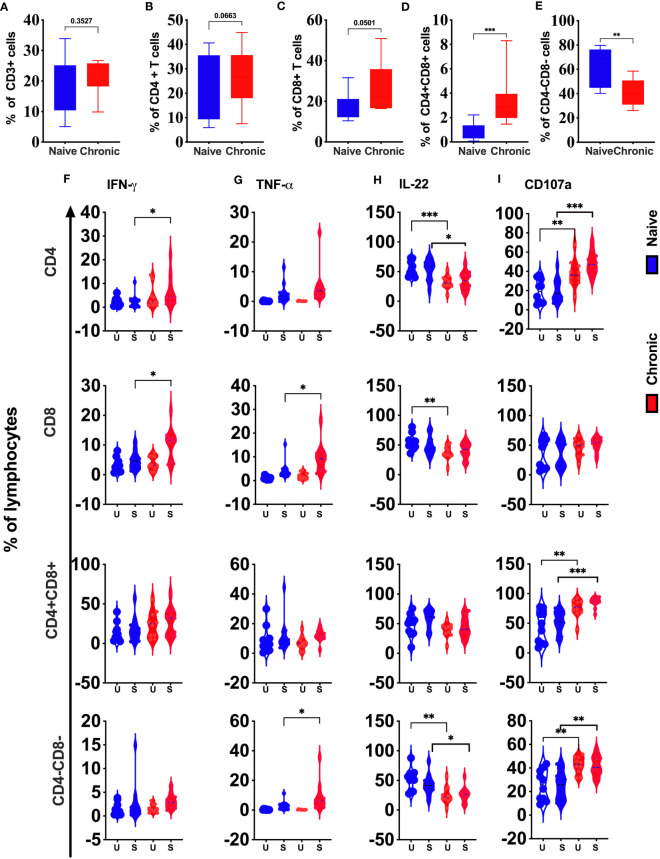
Phenotypic and functional comparison of tonsillar T cells in naïve and chronically SIV infected rhesus macaques. Frequencies of total T cells (CD3+) **(A)**, CD4+ **(B)**, CD8+ **(C)**, CD4+CD8+ **(D)**, and CD4-CD8- T cells **(E)** in naive and chronic SIV infected macaques. **(F)** IFN-γ, **(G)**TNF-α, **(H)** CD107a expression and **(I)** IL-22 production were measured in total T cells (CD3+), CD4+, CD8+, CD4+CD8+, and CD4-CD8- T cells in naive and chronic SIV-infected macaques. Mann–Whitney U-tests were used for naïve vs.-SIV comparisons and Wilcoxon matched pairs tests were used to compare NK cells and ILCs; *P < 0.05; **P < 0.01; ***P < 0.001.

Although peripheral blood T cell subsets show significant changes during chronic SIV infection, we did not find substantial differences in the frequency of total T cells (CD3^+^) (**Figure 6A**), CD4 T cells (**Figure 6B**) or CD8 T cells (**Figure 6C**) in tonsils between SIV infected and healthy animals. However, CD3^+^CD4^+^CD8^+^ T cells (double-positive T cells, **Figure 6D**) significantly higher in chronic SIV infection. In contrast, we found a significantly lower frequency of CD3^+^CD4^-^CD8^-^(DN) (**Figure 6E**) cells in infected tonsils. IFN-γ production was significantly enhanced in CD4 and CD8 T cells (**Figure 6F**) in chronic SIV infected animals. TNF-α was elevated in CD8 T cells and DN T cells (**Figure 6G**) from infected animals, while IL-22 secretion in unstimulated (baseline) was significantly lower in CD4 T cells, CD8 T cells, and CD4^-^CD8^-^ T cells (**Figure 6H**) and respectively in CD8 and DN T-cells after stimulation. Except for CD8 T cells, degranulation marker CD107a was elevated at baseline and after stimulation in CD4 T cells, CD3^+^CD4^+^CD8^+^ (DP) cells, and CD3^+^CD4^-^CD8^-^ (DN) T cells (**Figure 6I**) in infected animals.

### CD4^+^CD161^+^ T cells are significantly elevated during chronic SIV infection in the tonsils

**Figure 7:**
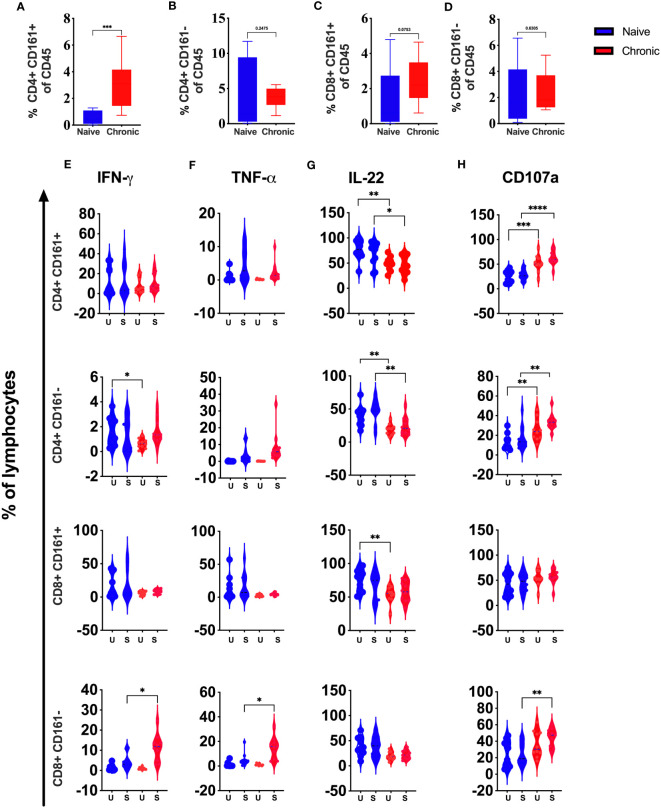
Role of CD161+ T cells during chronic SIV infection in rhesus macaques’ tonsils. Frequencies of CD4+CD161+T **(A)**, CD4CD161-T **(B)**, CD8+CD161+T **(C)**, and CD8CD161- T **(D)**, cells in naive and chronic SIV infected macaques. **(E)** IFN-γ, **(F)** TNF-α, **(G)** IL-22, **(H)** CD107a expression were measured in CD4+CD161+, CD4+CD161-, CD8+CD161+ and CD8+CD161- T-cells in naive and chronic SIV-infected animals after stimulation with PMA/Ionomycin. Mann–Whitney U-tests were used for naïve vs.-SIV comparisons and Wilcoxon matched pairs tests were used to compareNK cells and ILCs; *P < 0.05; **P < 0.01, ***p < 0.001.

Our high dimensional flow analysis suggested the presence of CD161^+^ T cells in macaque’s tonsils. These cell subsets have shown a role in chronic inflammatory conditions. However, in HIV infection, this phenotype was shown to be associated with high permissiveness for HIV-1 infection (16). We compared CD161^+^ and CD161^-^ CD4 and CD8 T cells in tonsils from SIV-infected and naïve animals. We found significantly elevated CD161^+^ CD4 T cells levels (**Figure 7A**) but not CD161^-^CD4 (**Figure 7B**), CD161^+^CD8 T cells (**Figure 7C**), or CD161^-^CD8 T (**Figure 7D**) cells. Secretion of IFN-γ and TNF-α, was not different between CD4^+^ CD161^+^ T cells from naïve, and SIV-infected animals. CD161^-^ CD4^+^ cells showed lower IFN-γ production at baseline with no difference for TNF-α (**Figures 7E, F**, respectively). CD161^-^ CD8 cells secreted more IFN-γ and TNF-α, post-stimulation compared to cells from naïve animals. However, no difference was seen for CD161^+^CD8 T-cells (**Figures 7E, F**). We found a general reduction (pre- and post-activation) of IL-22 in CD161^+/-^ CD4 T cell subsets from infected animals. Similarly, CD161^+^ CD8 T cell showed lower levels of IL-22 at baseline (**Figure 7G**) with no difference post-stimulation between infected and naïve animals. More CD161^+/-^ CD4 T cells from infected animals stained positive at baseline and post-stimulation for CD107a. No difference was observed for CD8^+^ CD161^+^ T cells, and CD8^+^CD161^-^ cells showed higher CD017a levels only after activation (**Figure 7H**).

## Discussion

Over the past few years, high dimensional flow cytometry advanced our understanding of immune cells in different tissues and organs. However, little is known about tonsillar immune cells and their role during HIV infection due to sample availability. Although there are few studies, most used tonsils collected in tonsillectomy due to underline pathology. Thus, healthy and HIV-infected tonsillar immune cell dynamics are still largely unknown. Our study used tonsils collected from healthy and chronic SIV infected macaques. Therefore, cellular dynamics in this study may represent pathological changes due to SIV infection.

A recent study by Hernandez ea. al.; characterized the NHP lingual tonsils using a basic immunophenotype panel and characterized T, B, and dendritic cell populations (17). However, this study was limited due to a lack of detailed immune cell markers. Compared to other studies, we perform extensive immunophenotype characterization of innate and adaptive immune cells in healthy tonsils. In addition, our study is the first comprehensive quantitative and functional description of innate and adaptive immune cell response during chronic SIV-infected and naïve tonsillar tissue.

We found that naive macaques tonsils dominated with a High frequency of CD45^+^CD3^-^CD20^-^NKp44^-^NKG2A^-^ (DN innate cells) cells followed by CD45^+^CD3^+^CD8^-^CD4^-^ (DN T cells) cells. However, Hernandez ea. al. did not study these populations in their analysis. In contrast to their report, we found that T and B cells were not significantly different, but B cells were slightly higher than CD3^+^ T cells. A similar observation was reported by Wohlford et.al (18)., in the human pediatric tonsils. In addition, similar to the human tonsils (19), we found higher CD4^+^T cells than CD8^+^T cells in the tonsils. We also saw more elevated classical monocytes, NKT-like cells in macaque tonsils. Finally, similar to macaque’s mucosal tissues (20), we found the NKB-like cell population in macaque’s tonsils.

Although previous studies compared individual tonsillar T, NK, and B cells separately in HIV^+^ humans and SIV^+^ animals, a comprehensive immune cell subsets dynamic analysis still needs to be done. Thus, using 27 different immune markers, we extensively studied innate and adaptive immune cell changes during chronic SIV infection in this study. Here we found significant alteration of innate and adaptive immune cell clusters in the unbiased clustering of tonsillar immune cells between healthy and chronic SIV infected animals.

Studies have shown that monocytes differentiate into DC or macrophages in tissues based on the tissue microenvironment. However, we found all three monocyte populations in the naive tonsils with similar proportions (21) to the human and macaque blood monocytes. Although the total monocyte frequency was not altered, we found a significant loss of inflammatory monocytes and higher classical monocyte subset in chronic SIV infected macaques. In addition, degranulation markers were significantly elevated in all three subsets in chronic SIV infected animals. Contrary, we previously found that blood monocyte subsets significantly higher in chronic SIV infection. This discrepancy may be due to the difference in the anatomical locations (22). However, inflammatory marker TNF-α was only elevated in non-classical and intermediate monocytes in SIV-infected animals. This may be due to the differentiation of inflammatory monocytes to macrophages (23) in an inflammatory environment like the tonsils in chronic SIV infection.

This observation warrants additional investigation. However, hypothesis is supported by previously published studies on the behavior of monocytes under different inflammatory conditions (21, 24–26).

Previous study has suggested that the oral mucosal NK cells play an important role in HIV/SIV infection (9). Thus, using our previously published gating strategy for mucosal NK cells, we found no significant differences between cytotoxic NK cells (NKG2A^+^ NK cells) and regulatory NKp44^+^ NK/ILCs in chronic SIV-infected macaques compared to naive animal tonsils. However, our data contradicted Li et al. (9), who reported elevated NKp44^+^ ILCs in macaque tonsils in chronic SIV infection. This may be due to the limited number of animals (n=4) used by Li et al.’s in their study. In addition, although we did not see a difference in the frequency of NKG2A^+^ NK and NKp44^+^ NK/ILCs in chronic SIV tonsils, we and others previously found significantly elevated NKG2A^+^ NK cells and depleted NKp44^+^ NK cells (10, 27) in the gut mucosal tissues of the chronic SIV infected macaques. Anatomical and physiological differences between tissues may contribute to these findings. However, like gut NKp44^+^ NK cells, tonsillar NKp44^+^ NK cells show higher production of CD107a, a cytotoxic marker. This may indicate the functional alteration of regulatory NK cells during chronic SIV infection. A similar observation was reported by Li et al. in tonsillar NKp44^+^NK cells (9). In addition, we found cytotoxicity of the CD3^-^CD20^-^CD14^-^NKp44^-^NKG2A^-^ (DN) population higher in chronic SIV infected animal’s tonsils. We previously found that the cytotoxic DN subset in gut mucosal tissues correlates with an increased risk of SIV acquisition (28). While this cell subset may be important for SIV pathogenesis in the gut and tonsillar tissues, the exact role of those cells in SIV pathogenesis remains to be elucidated. Peripheral blood CD3^+^CD56^+^(NKT-like) cells have been identified as a significant immune cell subset in response to viral infection and tumors (29–32). A recent study has shown that this subset is vital in slowing HIV disease progression (29). We found that tonsillar NKT-like cells significantly higher in chronic SIV-infected animals. Taken together, our data indicated that NKT-like cells might play a role in SIV pathogenesis in oral mucosal lymphoid tissues.

Chronic HIV/SIV infection is associated with an overall loss of CD4^+^T cells in the blood and gut mucosal tissues. However, Rosok et al. have shown that CD4^+^T cell count in asymptomatic HIV-1-positive individuals is similar to those observed in uninfected controls (33). Our study is consistent with these findings. In addition, although insignificant, we found elevated levels of CD8^+^T cells in chronic SIV infected macaques. Recently published research has shown significantly elevated CD8^+^T cells in the tonsils of people living with HIV (PLWH), supporting our macaque’s data. We also compared two unconventional T cell subsets, CD4^+^CD8^+^T cells (DP) and CD4^-^CD8^-^T cells (DN), between naive and chronic SIV^+^ macaques. DP T cells represent a heterogeneous population. However, evidence indicates that *in vivo* terminally differentiated effector CD4^+^ may acquire the α-chain of CD8. DP T cells have been higher in HIV (34, 35) and other viral infections (36, 37) autoimmune and chronic inflammatory disorders (38, 39). Here we report significantly higher DP T in chronic SIV infected macaques’ tonsils. We also notice enhanced cytotoxicity measured by elevated CD107a in those cells during chronic SIV infection. This may suggest DP T cells may be important in the fight against HIV infection. Compared to blood (40, 41), our data shows CD3^+^CD4^-^CD8^-^ DN T cells are the dominant CD3^+^cells in macaques’ tonsils for the first time. Other studies have suggested that DN T participates in inflammatory diseases and viral infections, including HIV (42, 43). Contrary to these other studies, we found a significant loss of DN T in the tonsils during chronic SIV infection. In addition, we also observed higher cytotoxicity and TNF-α production during chronic SIV infection. However, the exact role of DN T cells in tonsils during SIV infection is unclear. CD161 is a C-type lectin-like receptor expressed on natural killer (NK) cells; however, it was shown that CD161 is expressed on subsets of CD4^+^ and CD8^+^T cells (44–48). Li et al. found that CD161^+^CD4^+^ T cells are highly permissive to HIV-1 infection, and they further showed that the cells harbor more replication-competent latent HIV-1 in blood and lymph nodes (16). In this study, we show for the first time, that CD161^+^CD4 T cells are significantly higher in chronic SIV-infected macaques tonsils. This may suggest that macaque’s tonsils harbor latent HIV reservoirs in this T cell subset. However, further studies are needed to confirm this observation. Although we expected significant changes in the B cells subset in tonsils during chronic infection, we did not see any substantial changes in total B cells. Unfortunately, we could not identify differences in B cells subsets as we did not have specific markers in our flow cytometry panels.

Limitations of our study. We have used our archived tonsillar mononuclear cells from our previous pathogenesis study (10). Thus, the number of cells was limited and didn’t yield enough material to quantify SIV DNA/RNA in immune cells and transcriptional profiling of naive and chronic tonsillar immune cells. All these limitations warrant further detailed investigation.

In summary, we describe innate and adaptive immune cell populations found in the non-human primate tonsils and describe changes during chronic SIV infection. We showed that naive macaques’ tonsils are dominated by innate immune cells, followed by B cells and T cells; we show for the first time that significant alterations of classical and intermediate monocytes, functional changes of NK/ILCs subsets and other innate immune cells, DP T, DN T cells and CD161^+^CD4 T cells during chronic SIV infection in macaques’ tonsils. Taken together, our results show the fundamental changes during chronic SIV infection in the tonsils. However, more work is needed to investigate whether these changes can lead to HIV-associated oral lesions and malignancies in the tonsils and oral mucosal tissues.

## Data availability statement

The raw data supporting the conclusions of this article will be made available by the authors, without undue reservation.

## Ethics statement

The study was reviewed and approved by the animal care and use committee at Advanced Bioscience Laboratories (Rockville, MD).

## Author contributions

Study conception and design: NL. Coordinated and performed the study: RS and MG with NR-L. MD, AH, and SC. Analysis and interpretation of data: RS, MG, NR-L, MD, AH, SC, KW, DK, NL, and TD. Drafting of the manuscript: RS, MG, NL, and TD. Performed statistical analysis of the data: KW and DK. The first author order was determined by relative effort in data acquisition and analysis, drafting of the manuscript, and overall workload. All authors read and approved the final manuscript.

## Supplementary material

Supplementary Figure 1 Representative gating strategy for identifying innate and adaptive immune cells in Macaque tonsillar tissue.

Supplementary Figure 2 B cells during chronic SIV infection in rhesus macaques’ tonsils. Frequencies of CD20 cells in naive and chronic SIV infected macaques.

Supplementary Figure 3 Representative plots showing cytokine production and CD107a expression on T cell subsets.

Supplementary Figure 4 Representative plots showing cytokine production and CD107a expression on NK cell subsets.

Supplementary Figure 5 Representative plots showing cytokine production and CD107a expression on Monocytes subsets.
