# Supplementary figures and images for: Innate adaptive immune cell dynamics in tonsillar tissues during chronic SIV infection
Source: Front Immunol. 2023 Aug 21;14:1201677. doi: 10.3389/fimmu.2023.1201677 (PMC10475724; doi:10.3389/fimmu.2023.1201677)

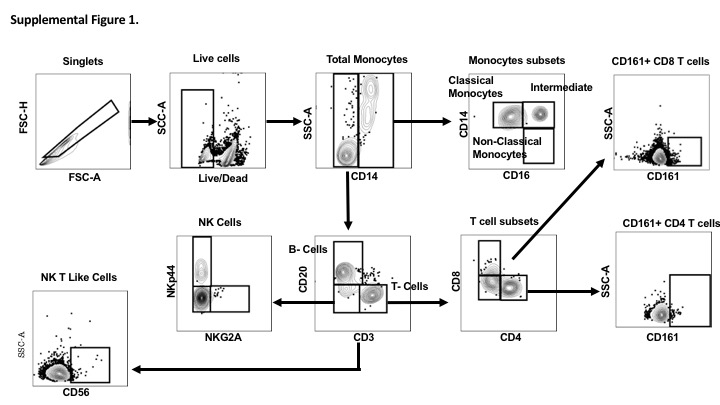

Supplement: Supplementary Figure 1 — Representative gating strategy for identifying innate and adaptive immune cells in Macaque tonsillar tissue. [file Image_1.jpeg]

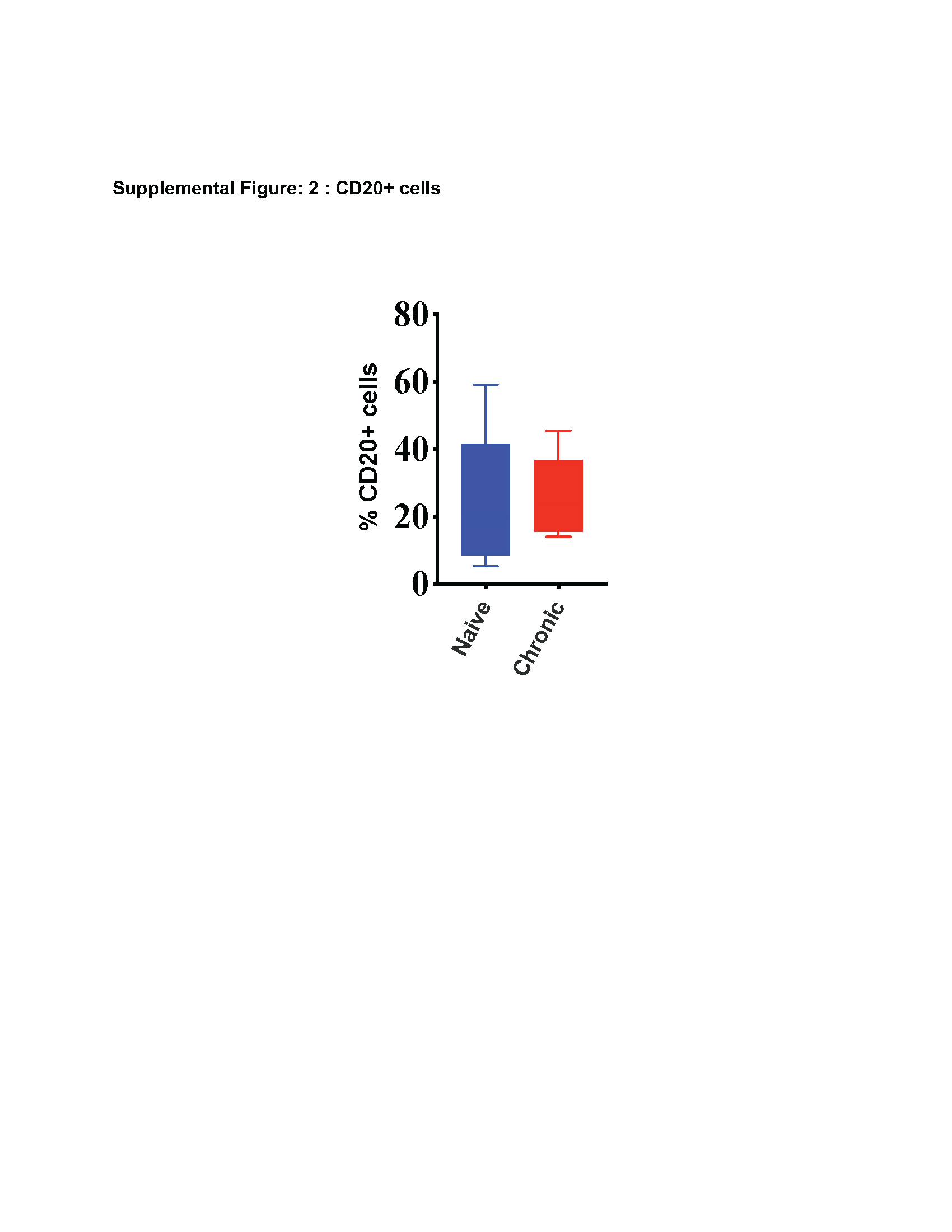

Supplement: Supplementary Figure 2 — B cells during chronic SIV infection in rhesus macaques’ tonsils. Frequencies of CD20 cells in naive and chronic SIV infected macaques. [file Image_2.jpeg]

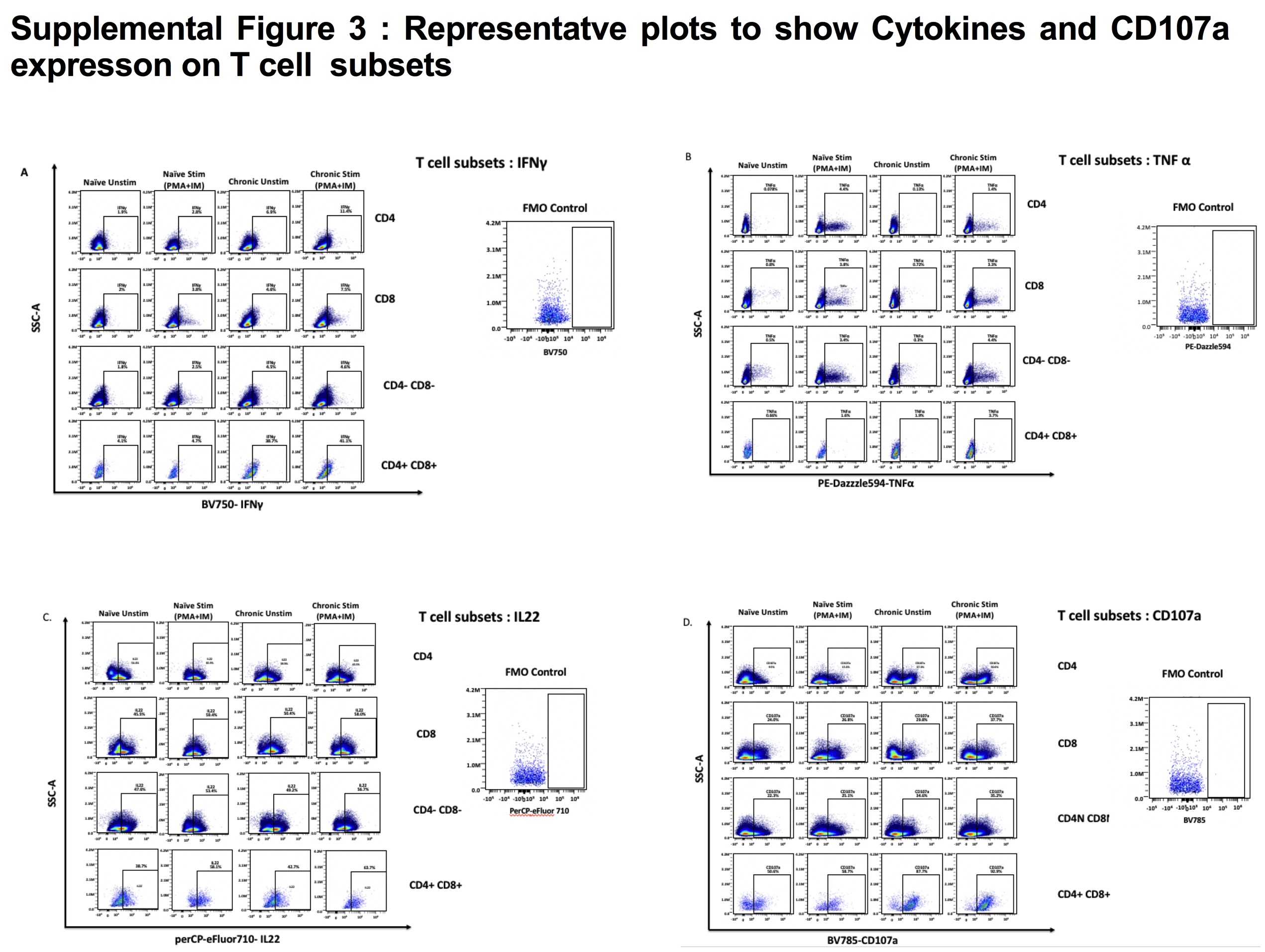

Supplement: Supplementary Figure 3 — Representative plots showing cytokine production and CD107a expression on T cell subsets. [file Image_3.jpeg]

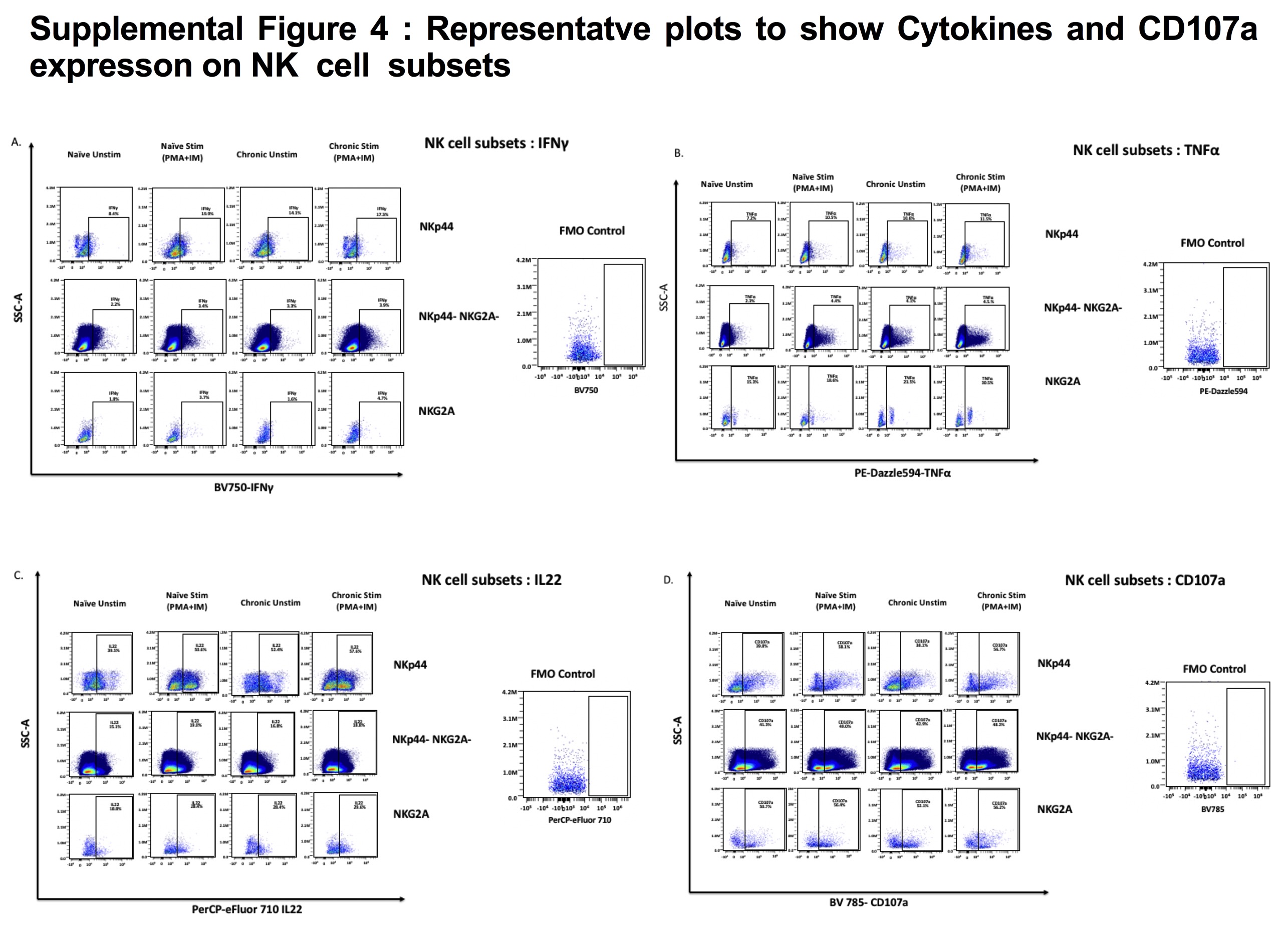

Supplement: Supplementary Figure 4 — Representative plots showing cytokine production and CD107a expression on NK cell subsets. [file Image_4.jpeg]

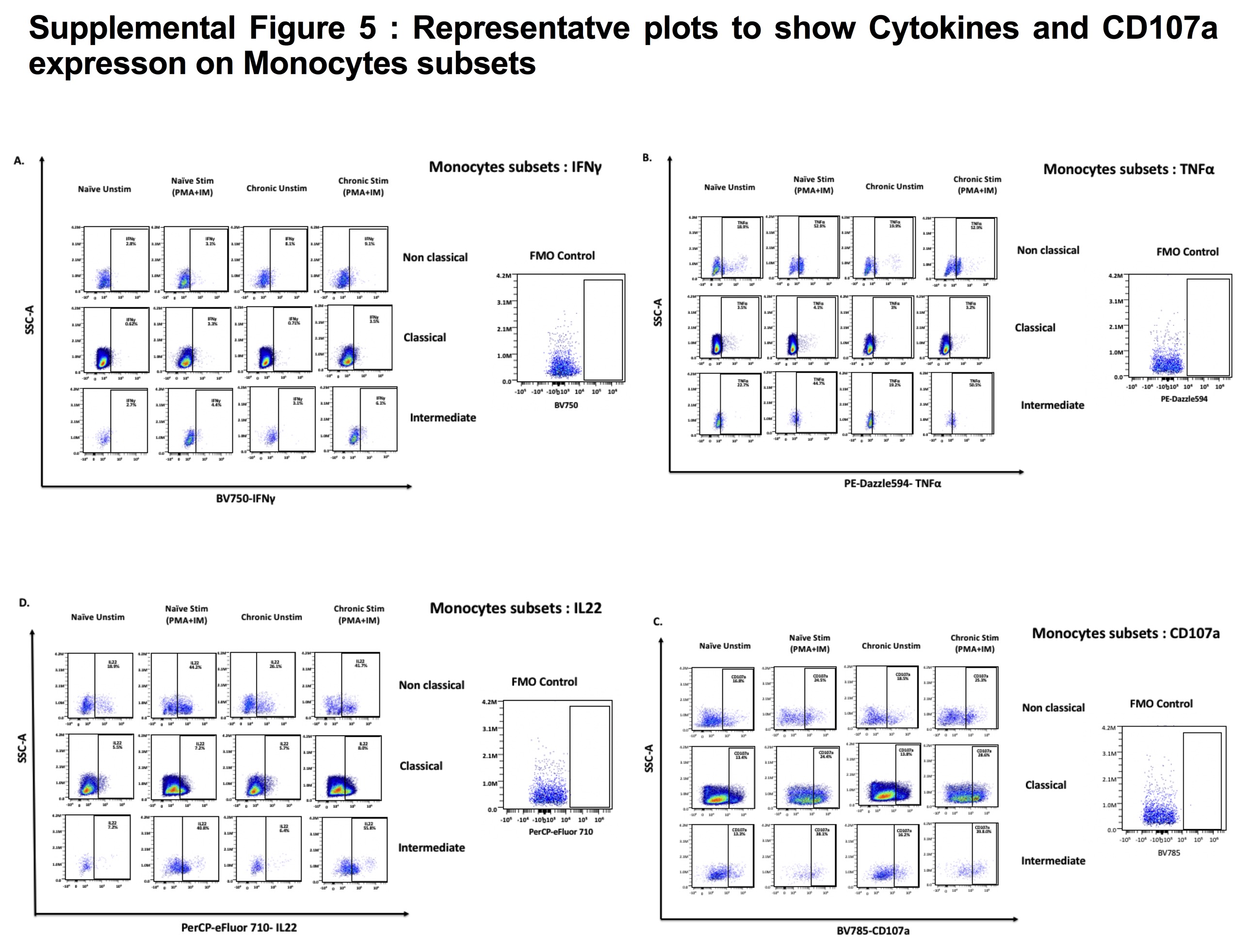

Supplement: Supplementary Figure 5 — Representative plots showing cytokine production and CD107a expression on Monocytes subsets. [file Image_5.jpeg]
