# Supplementary material for: Innate adaptive immune cell dynamics in tonsillar tissues during chronic SIV infection
Source: Front Immunol. 2023 Aug 21;14:1201677. doi: 10.3389/fimmu.2023.1201677 (PMC10475724; doi:10.3389/fimmu.2023.1201677)
Supplement: Supplementary file 6 [file Table_1.docx]

Table 1: List of antibodies for Flow Cytometric analyses.

| S.No | Antibody | Clone | Fluorochrome | Manufacturer | Catalog No |
| --- | --- | --- | --- | --- | --- |
| 1 | Anti-CD-16 | 3G8 | Pe-Cy-5 | BD | 555408 |
| 2 | Anti-CD-4 | SK-3 | Super bright | Thermo Fisher | 62-0047-42 |
| 3 | Anti-CD-8 | RPA-T8 | AF532 | Thermo Fisher | 58-0088-42 |
| 4 | Anti-CD-3 | SP34-2 | APC-Cy-7 | BD | 557757 |
| 5 | Live/Dead |  | Aqua | Thermo Scientific |  |
| 6 | Anti-CD20 | 2H7 | Brilliant Violet -605 | Biolegend | 302333 |
| 7 | Anti-NKP-44 | P44-8 | PE-CY-7 | Biolegend | 325116 |
| 8 | Anti-CD45 | DO58-1283 | FITC | BD | 557803 |
| 9 | Anti-CD-127 | A019D5 | Brilliant Violet -421 | Biolegend | 351310 |
| 10 | Anti-CD27 | O323 | Brilliant Violet -570 | Biolegend | 302825 |
| 11 | Anti-CD159a | Z199 | PE (B5) | Beckman Coulter | IM3291U |
| 12 | Anti-CD56 | HCD56 | Brilliant Violet -711 | Biolegend | 318336 |
| 13 | Anti-CD14 | M5E2 | Pacific Blue (V3) | BD | 558121 |
| 14 | Anti-CRTH-2 | 301109 | AF-647 (R2) | R and D (fisher) | FAB33381G |
| 15 | Anti-CD117 | 104D2 | Brilliant Violet -650 | Biolegend | 313222 |
| 16 | Anti-CD107a | H4A3 | Brilliant Violet-785 | Biolegend | 328644 |
| 17 | Anti-CD161 | 561 | AF700 ( R5) | Biolegend | 343621 |
| 18 | Anti-IL-17a | eBio64DEc17 | Per-CP-CY5.5 (B9) | eBioscience | 45-7179-42 |
| 19 | Anti-IL-22 | IL22JOP | PerCP-eFlour-710 | eBioscience | 46-7222-82 |
| 20 | Anti-TNF-α | MAb11 | PE-DAZZLE-594 | Biolegend | 502945 |
| 21 | Anti-IFN- γ | B27 | Brilliant Violet -750 | BD | 566357 |
